# Medical Opinion and Movement

**Published:** 1908-02-22

**Authors:** 


### Medical Opinion and Movement.

Since Rehn first successfully sutured the human heart eleven years ago, wounds of the heart have definitely come within the province of the surgeon, and considerable advances have been made in the treatment of such lesions. Within this period about 120 similar operations have been performed, of which 46 per cent. have recovered. It has been shown by Fischer and Loison that only 15.63 per cent. of cases not treated by operation have recovered. Felix Franke considers that by improved methods and early diagnosis the mortality should be further reduced. It has been ascertained that in the majority of fatal cases death is due to compression of the heart by blood in the pericardium rather than to loss of blood. Franke, therefore, lays stress on the importance of paracentesis in the treatment of cardiac wounds. Puncture may only serve as a palliative means, and it may still be necessary for the surgeon to operate and suture the wound; but when the symptoms are urgent, it may act as a life-saving treatment, and in some cases may be in itself sufficient. As a rule, the fifth intercostal space, close to the sternum, is the safest place for punctures, but the extent of the dulness must be used as a guide to determine the position for puncture. Franke is of opinion that by a freer use of the puncture of the pericardium the mortality from wounds of the heart may be reduced.

The efficacy of lumbar puncture in acute meningeal infections is already well established. Dr. Ch. Rocaz and Dr. Firmin Carles have recently reported on eight cases of enteric fever in children in which meningeal complications were present and in which lumbar puncture was successfully employed. In these, as in other cases, lumbar puncture has not only proved of therapeutic value, but it has also been a valuable means of diagnosis and of elucidating the pathology underlying these conditions. These authors have been able to distinguish clinically four types of meningeal manifestations in enteric fever in children, and have found corresponding variations in the condition of the cerebro-spinal fluid. The first type is distinguished by intense headache, repeated vomiting and constipation, irregularity of pulse and respiration, various vasomotor disturbances, cutaneous hyperæsthesia, and, finally, retraction of the head. In the second type the complete tableau of acute cerebro-spinal meningitis is presented. Kernig's sign appears, rigidity of the neck and spine follows, and this rigidity may extend to the limbs. The third type is rarer and presents from the first the clinical picture of tuberculous meningitis. The fourth type is peculiar to infants and consists of convulsions which lead to a rapidly fatal termination, corresponding to variations in the severity of the symptoms. Lumbar puncture may yield pus with typhoid bacilli in pure or mixed culture; a turbid or transparent fluid with typhoid bacilli or others; a clear fluid with abundant lymphocytes; or, finally, a fluid of normal composition, but under high pressure. In all these conditions lumbar puncture is efficacious by removing pus, microbes, and accumulated toxins, or simply by relieving the pressure due to excessive secretion of cerebro-spinal fluid.

It is now generally accepted that tuberculosis in man arises from two types of tubercle bacillus, the typus humanus and typus bovinus, and an endeavour has been made to classify tuberculous lesions according to the type of bacillus which is responsible for them. On various grounds the opinion has been formed that tuberculosis of the respiratory system is caused by tubercle bacilli of the typus humanus, and that bacilli of the typus bovinus give rise to tuberculous lesions of the peritoneum, joints and glands, lesions which are especially common in childhood. Adopting these views, Dr. Nathan Raw has further been led to the conclusion by his observations and research, that Koch's tuberculin R, prepared from human tubercle bacilli, is only efficacious in cases of bovine tuberculosis, whilst phthisis pulmonalis requires a tuberculin prepared from bovine sources. This interesting conclusion has been put to practical test and has so far yielded some striking results. It is already recognised by many observers that injections of Koch's tuberculin are much more efficacious in cases of so-called "surgical" tuberculosis (now identified with bovine tuberculosis) than in cases of pulmonary disease. Dr. Nathan Raw has treated 104 cases of surgical tuberculosis with Koch's tuberculin, including 23 cases of glands, 24 cases of lupus, 27 cases of tuberculous joints, 8 cases of tuberculous peritonitis, and 4 cases of meningitis, and is most enthusiastic in regard to the results of the injections. All the cases of lupus healed rapidly with so far only two recurrences. For the treatment of phthisis pulmonalis Dr. Raw has prepared a serum from tuberculous cattle, and he has at the present time 16 cases under treatment. From the account he gives of the results of the treatment thus far the injections appear to have had a marked effect upon the course of the disease in several of the cases, and his further results will be awaited with interest.

Dr. Emil Weil, of Paris, has attempted to combat hæmophilia and allied conditions by treatment with intravenous and subcutaneous injections of fresh animal serum. He worked on the theory that these conditions were due to a deficiency in coagulation power, and sought to remedy this by an artificial supply of fibrin ferment. Following on these lines, Mr. E. C. Hort has obtained better results by administering much larger doses of serum by the mouth. By extending the range of application of this kind of treatment he has found remarkable improvement follow in cases of many affections in no sense hæmorrhagic, such as chlorosis and gastric ulcer. He has published accounts of a few of these cases, which appear to give clear evidence of a very markedly favourable effect of the treatment on the course of the disease. In conditions of chlorosis severe symptoms of dyspepsia, dyspnœa, and such like have disappeared, and blood counts have shown a rapid increase in the number of red corpuscles and hæmoglobin content. Treatment of cases of tuberculous hæmoptysis and duodenal ulcer with hæmorrhage has yielded similarly favourable results. Mr. Hort, together with several other collaborators, is now engaged in further researches, both in the laboratory and in the wards of several hospitals, on this treatment of morbid conditions by normal serum, in order to discover the limits and conditions of the treatment, and, if possible, the principles underlying it. He suggests that the administration of normal serum may affect the production of auto-anticomplements, or in some other way correct a disturbance of the regulating mechanism. Gastric ulcer, for instance, may be a local expression of a general blood dyscrasia, and due to the presence in the blood of cytolysins for gastric epithelium. Such hypotheses are at present pure speculation. Further investigations alone will be able to establish this normal serum therapy on a rational basis, and demonstrate its medicinal value.

The Calmette-Eisnerr ophthalmo-tuberculin reaction, of which so much has been heard lately, is the subject of an interesting article by Dr. Poulard in *Progres Médical.* The writer lays stress on the unreliability of the reaction in cases of advanced tuberculosis—a fact already pointed out by previous observers—and concludes that though in many cases a negative reaction is of great assistance in clearing up an obscure diagnosis, a positive result is hardly of much value in a case which is on the borderland. He draws attention to the dangers and inconveniences resulting from the employment of tuberculin applications to the conjunctivæ. In one case he has seen the inflammatory reaction persist for several weeks and give rise to corneal lesions. Where there is reason to suspect tubercle of the uveal tract this method of diagnosis should in no case be used.

Kuttner (*Deutsch. Med. Wochenschrift*, No. 43, 1907) reports his experience in the treatment of cases of chronic diarrhœa. Such cases are due to (1) chronic irritation of the alimentary canal, especially the intestine, (2) nervous causes, and (3) blood conditions. As a type of the first category entozoic diarrhœa due to the presence of ascarides may serve; of the second the chronic diarrhœa occurring in cases of exophthalmic goitre, and the so-called "functional" diarrhœas occurring in certain cases of chronic intoxications, such as morphinism, chronic alcoholism, etc.; while finally as a type of the third class the diarrhœa met with in some cases of uræmia, malaria, and septicæmia may be cited. The author goes very fully into the differential diagnosis, and lays stress on the necessity for careful examination of the fæces in each case. In the treatment he recommends attention to diet and scrupulous consideration of each individual case. He cautions against the indiscriminate use of astringents, and recommends care in the administration of the most useful remedy the physician possesses in the treatment of these cases—namely, opium.

"Neuritis Complicating Hepatic Cirrhosis" is the subject of an interesting paper by Drs. Klippel and L'Hermitte. The authors consider multiple neuritis by no means a rare affection in cases of advanced cirrhosis. They conclude that it has not been proved that in these cases alcohol is the primary and sole cause of the affection, which by some authors is held to be the case. On the contrary, they incline to the view that the neuritis is due to an auto-intoxication dependent upon hepatic insufficiency. Histologically, the nerves show evidence of parenchymatous lesions. Clinically, the affection is characterised by a rather sudden onset, paralysis, trophic troubles, and by a rapid course, death usually supervening within a short time after the onset. The appearance of marked symptoms of neuritis in a case of cirrhosis, whether alcoholic or otherwise, is therefore considered of grave prognostic significance.

Lenz, in operating on a patient for intestinal obstruction, had occasion to resect a large portion of the mesocolon, and determined to replace the mesentery by a piece of omentum. He had previously experimentally substituted in dogs for large pieces of excised mesentery, portions of the large omentum. In his recently published report he gives details of his case. The patient was a woman, aged 60 years, admitted for intestinal obstruction, diagnosed as due to malignant growth at the splenic flexure. At the operation a volvulus was found, and when this had been rectified a small growth was discovered at the pylorus. This was resected, and as it was found that the transverse mesocolon was largely adherent to it, an extensive resection was undertaken, the middle colic artery being ligatured. To prevent necrosis of the colon the large omentum was brought into contact with the intestinal edge of the mesocolon and sutured on to the colon. The patient made an excellent recovery.

An interesting point has recently attracted the attention of certain French observers. This is the antiseptic properties of wines when taken internally. Carles, at Bordeaux, has made a series of experiments to investigate the theory that the use of wines to dilute water suspected of impurity will be a safeguard against enteric fever. His conclusions are extremely interesting. White wines were found to have an appreciable bactericidal action, which is quite independent of the amount of alcohol they contain, since the bactericidal properties are not destroyed on heating the wine. This bactericidal power of white wines appear to depend on their acidity, since the use of an alkali entirely destroys it. The author concludes that there is decided justification for the belief that mixing white wine or acid red wine with water suspected of impurity will be of service in destroying bacteria that may be present in such water. He advised that the mixture of wine and water should be made several hours before it is required for use, and thinks that this method may usefully be tried in cases where it is impossible, for various reasons, to sterilise the water by boiling.

Luys, in *La Clinique*, January 10, 1908, deals with the diagnosis of renal tuberculosis. Two questions have to be answered by the medical attendant in each suspected case: (1) Is renal tuberculosis present? and (2) if so, is it unilateral or are both kidneys involved? The author lays stress on the opinion that in unilateral renal tubercle prompt nephrectomy is necessary, provided the other kidney is functioning properly. In establishing the diagnosis very careful examination of the urine (twenty-four hours' specimen) should be a routine measure, accompanied by the usual bacteriological tests. In the physical examination particular attention should be paid to what Luys calls the "ureteric-vesical reflex"—a dull pain over the vesical end of one or other ureter. To prove the functional activity of the other kidney, or rather to exclude as thoroughly as possible disease in it, cystoscopic segregation and thorough examination of the separated urines are strongly recommended.

Dr. Carl Leuwer, of Bonn, in a clinical lecture published in a recent number of the *Reichs Medizinal Anzeiger*, offers some interesting notes on a series of cases of Vincent's angina (or Plaut-Vincent's disease, as it is called in Germany). The importance of differentiating this condition, which is by no means a rarity in England, from diphtheria is well recognised, but although Miller, as early as 1883, described the specific organism, few or our text-books devote sufficient attention to the malady. The main symptoms of a typical case of Vincent's angina are those of tonsillitis. The submaxillary glands are usually affected, and the constitutional disturbance is generally severe. On examination one tonsil is found affected, and this is usually the right one. There is definite ulceration, and sometimes membrane, which is hard to distinguish by the naked eye from diphtheritic membrane. The diagnosis lies between Vincent's angina, diphtheria, syphilitic ulceration, and acute follicular tonsillitis. A smear preparation taken from the affected gland will at once clear up the diagnosis. The slide is stained as for tubercle, and the specific organisms are usually readily distinguished as short, acutely pointed rods, more or less spirally twisted. They take the stain well, and are differentiated from the Klebs bacillus by the fact that they are much larger and pointed, and that they cannot be cultivated on the ordinary media. The diagnosis from syphilitic ulceration is sometimes difficult but may be made by paying attention to the concomitant signs. The prognosis in a case of Vincent's angina is usually very good. The disease runs its course in two or three weeks, usually with no complications. All that is necessary in treatment is to keep the patient in bed and to support his strength by suitable tonics, diet, etc., and to treat the local condition, preferably, according to the author, by means of a weak (1 per cent.) solution of corrosive sublimate.

Professor Calmette, in a paper read before the Paris Academy of Medicine at its last meeting, urged a wider employment of what is known as the "Calmette-Eissner Opthalmo-Tuberculin Reaction." As a means of diagnosis, he thought the reaction should be widely known. It was an easy, safe, and comparatively delicate reaction, within reach of every general practitioner, and he advocated its routine use, as a diagnostic measure, in the case of public servants, school children, soldiers and sailors. Cases where, on examination, a positive reaction was given were at least presumably tuberculous and should be placed under strict surveillance. It was the best means of selection and its ease and safety made it an almost ideal diagnostic help. In the discussion that followed the reading of the paper, Professor Delorme, who has made great use of this test, drew attention to the equivocal results sometimes obtained. The reaction was not constant in infants, and sometimes failed in very much debilitated subjects. The systematic use of the reaction was attended with some difficulties, especially so far as its routine employment in the Army was concerned. A positive reaction was undoubtedly of great value, but a negative result certainly did not exclude tubercle. This has been the first important discussion on what promises to be a much used means of diagnosis in the future.

At the Neurological Congress, held at Washington in 1888, Dercum presented a case of a woman suffering from a condition which in many ways appeared to resemble myxœdema, but which differed in several minor points from this well-known disease. Since that time "Dercum's disease" has been looked upon as a rarity, and accounts of it are not very frequently to be found in text-books. It is therefore interesting to notice that this "syndrome of Dercum" has been made the subject of a close study in a thesis by Dr. E. Pinet, of Lille. "Dercum's disease" is a condition of diffuse lipomatosis, accompanied by definite painful symptoms, and usually associated with a peculiar mental condition which is almost characteristic. All the subjects so far have been women. Alcoholism, trauma, epilepsy, hysteria, syphilis, the menopause, emotional excitement, heredity climatic conditions, tubercle and influenza have all been assigned as etiological factors. Probably none of them has anything to do with the disease. Two autopsies have been made by Dercum himself. In both signs of interstitial neuritis were found, with atrophy of peripheral nerves, and in one case slight degeneration in the postero-mesial column of the cord. All the patients are exceedingly fat. They complain of dull pains, of general lassitude, and may later on in the course of the malady be subject to trophic disturbances. The differentiation between this syndrome and that of myxœdema, with which it is most likely to be confounded, is chiefly made by attention to the following points: in myxœdema the face, hands and feet are involved in the general enlargement; in Dercum's disease these parts are more or less normal. Pain is never complained of in myxœdema; in this condition it is more or less constant. In myxœdema, too, the mental faculties are dulled, while in Dercum's disease they are unimpaired. In the latter condition thyroid extract has no influence upon the obesity. The author concludes that cases of Dercum's disease are by no means rare, but that they are rarely diagnosed.

